# Development of Thermally Conductive Polyurethane Composite by Low Filler Loading of Spherical BN/PMMA Composite Powder

**DOI:** 10.1038/s41598-019-50985-5

**Published:** 2019-10-07

**Authors:** Kai-Han Su, Cherng-Yuh Su, Cheng-Ta Cho, Chung-Hsuan Lin, Guan-Fu Jhou, Chung-Chieh Chang

**Affiliations:** 1 Institute of Mechatronic Engineering, National Taipei University of Technology, 1, Section 3, Zhongxiao E. Rd., 106, Taipei, Taiwan; 2 Additive Manufacturing Center for Mass Customization Production, National Taipei University of Technology, 1, Section 3, Zhongxiao E. Rd., 106, Taipei, Taiwan; 3 GUS Technology, New Taipei City, 22175 Taiwan

**Keywords:** Mechanical engineering, Composites

## Abstract

The issue of electronic heat dissipation has received much attention in recent times and has become one of the key factors in electronic components such as circuit boards. Therefore, designing of materials with good thermal conductivity is vital. In this work, a thermally conductive SBP/PU composite was prepared wherein the spherical *h-*BN@PMMA (SBP) composite powders were dispersed in the polyurethane (PU) matrix. The thermal conductivity of SBP was found to be significantly higher than that of the pure *h-*BN/PU composite at the same *h-*BN filler loading. The SBP/PU composite can reach a high thermal conductivity of 7.3 Wm^−1^ K^−1^ which is twice as high as that of pure *h*-BN/PU composite without surface treatment in the same condition. This enhancement in the property can be attributed to the uniform dispersion of SBP in the PU polymer matrix that leads to a three-dimensional continuous heat conduction thereby improving the heat diffusion of the entire composite. Hence, we provide a valuable method for preparing a 3-dimensional heat flow path in polyurethane composite, leading to a high thermal conductivity with a small amount of filler.

## Introduction

In recent times, the electronic industries are incessantly focusing on the three major development trends: high performance, miniaturization, and integration in the electronic products. The issue of electronic heat dissipation has received much attention since the excessive heat generated by the electronic device increases the temperature of the component leading to thermal fatigue, which in turn influences the reliability and reduces the service life of the electronic device^1^. Therefore, it requires the heating element with low thermal resistance and applies the high-efficiency heat sink in the heat dissipation path of the electronic components. Moreover, the package joint of the interface between each material and the heat sink is also an important key for thermal management technologies.

Polymer-based composites blended with different fillers possessing high thermal conductivity can be the potential materials that are capable of satisfying the above requirements. The composite material consisting of two or more different materials with characteristics of individual constituent materials, for example, lighter weight, better electrical insulation, and good heat resistance, good material strength, and good processability^2,3^. To date, many studies have pointed out that metals and carbon-based fillers such as graphene can be used as additives to improve the thermal conductivity of polymer-based composites^4,5^. Zhang *et al*. studied the thermal transport in supported graphene on a *h-*BN substrate, and observed impressive thermal conductivity by stress controlled graphene^6^. Lu *et al*. prepared a three-dimensional hybrid hierarchical structure with carbon nanotube intercalated graphene sheets by thermal annealing of carbon nanotube/graphene oxide films. The hybrid films demonstrated ultra-high in-plane thermal conductivity because CNT forms a bridge, connecting the graphene sheets to improve the phonon propagation and avoid the corrugation of graphene layers during thermal treatment^7^. Although these classes of composite materials display excellent thermal properties as the thermal interface materials in electronic packaging, they possess high electrical conductivity, resulting in current leakage and electrical short-circuit. These problems limit the practical application of composite material in the electronic packaging that requires electrical insulation and therefore, ceramic particles are commonly used as thermally conductive fillers^8^. Among the ceramic additives, hexagonal boron nitride (*h-*BN) is one of the most suitable candidates for it is comparable to graphite in terms of structure and physical properties, and possesses high thermal conductivity, high-temperature resistance, low reactivity, electrical insulation, and excellent oxidation resistance. Comparing with other fillers, it has various advantages such as lighter weight and low cost^9–11^. The thermal conductivity of the composite material is mainly affected by the dispersion of the filler in the polymer matrix and the interfacial thermal resistance between the filler and the polymer matrix^12^. Many studies have focused on the surface modification of the filler and combined with different mechanical blending methods to increase the dispersion of the filler in the polymer matrix and enhance the thermal conductivity of *h*-BN. However, these methods cannot efficiently increase the thermal conductivity since the surface functionalization process is complicated. Furthermore, higher concentrations of fillers are required to form thermally conductive channels, resulting in increased costs of fillers and may reduce the mechanical properties of the composite. Therefore, it remains a challenge in achieving high thermal conductivity of the composite with low filler loading^13,14^. The *h-*BN has a layered structure; thus, it exhibits a higher thermal conductivity when the long axis of the *h-*BN composite is parallel to the heat transfer direction, while shows lower when the long axis of the *h-*BN composite is perpendicular to the heat transfer direction. This phenomenon is due to the heat transfer mechanism of phonon modes, and many studies demonstrate that the arrangement of *h-*BN additives in the polymer matrix and thermal properties are closely related^15–17^. Thus, forming a continuous three-dimensional heat conduction network along the heat transfer direction and combining with the tightly stacked *h*-BN structure uniformly dispersed in the polymer matrix will be the one of the most favorable strategies to achieve high thermal conductivity^18,19^.

Here we have found a simple, fast and effective method to prepare a compact and continuous *h-*BN heat conduction network in the polymer matrix. Firstly, spheroidized *h*-BN and Polymethyl methacrylate (PMMA) composite were prepared by mechanical mixing and spray drying process^20,21^. Then, the spherical composite powder was uniformly filled in the PU to prepare a thermally conductive composite material. The purpose of our study is to improve the thermal conductivity of *h-*BN/polyurethane (PU) composite by spheroidizing *h*-BN composite powders while taking into account heat transfer directionality and continuity. Through direct FE-SEM observation, dispersion analysis and interphase behavior investigation of spheroidized *h*-BN and spheroidized *h*-BN/polyurethane (PU) composites were measured and executed. Our results indicate that spheroidized SBP/PU composite show a uniform dispersion of the filler in the polymer matrix and an ordered thermal network with a high thermal conductivity of 7.3 Wm^−1^ K^−1^ containing 40 wt% filler loading. The composite material prepared by this method possesses high thermal conductivity and also overcomes the poor dispersion of *h-*BN in the polymer matrix along with the stringent requirement of orientation for heat transfer in *h-*BN. This method is facile to execute, and it can also decrease the amount of *h-*BN additives used and in turn, greatly reduce the material cost.

## Results and Discussion

**Figure 1:**
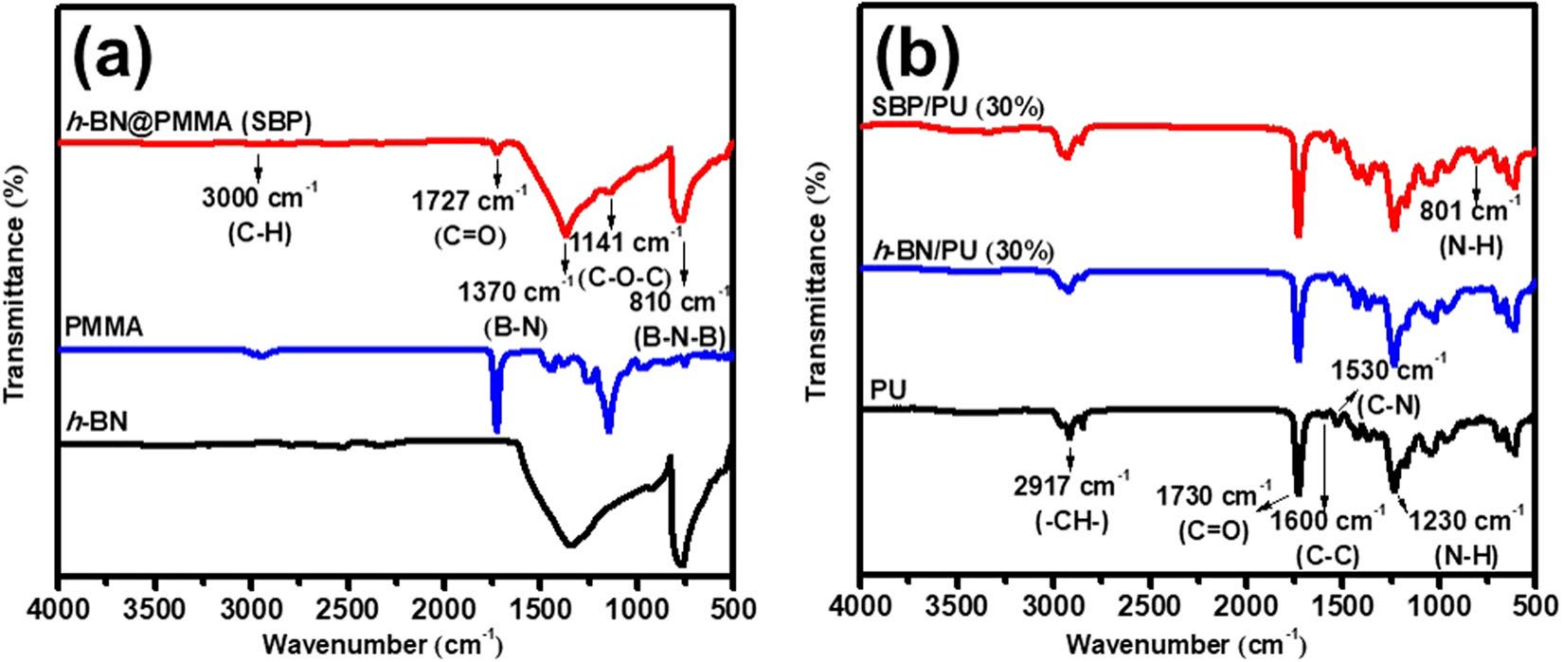
FTIR spectrum of (**a**) pure *h-*BN, PMMA and SBP powders and (**b**) pure PU, *h-*BN/PU and SBP/PU composites with 30 wt% filler loading, respectively.

The prepared SBP was dispersed in the PU matrix to form the high thermally conductive composite wherein the FTIR was utilized in understanding the formation of the composite (30 wt% of filler loading) with the preliminary identification of their chemical structures. Figure 1(a) shows the FTIR spectrum of *h*-BN powder, PMMA, and *h*-BN@PMMA (SBP). The pure *h-*BN exhibits two characteristic peaks at the wavenumber of 1370 cm^−1^ and 810 cm^−1^, indicating B-N in-plane stretching mode and B-N-B out-of-plane bending mode, respectively^22^. From the spectrum of pure PMMA, there are three characteristic peaks that can be observed at wavenumber of 1141 cm^−1^, 1727 cm^−1^ and 3000 cm^−1^. These peaks correspond to C-O-C single bond stretching mode, carbonyl group (C=O) and C-H bond stretching mode^23^. The FTIR spectrum of SBP is dominated mainly by the characteristic BN peak at 1370 cm^−1^, however, also contains the carbonyl peak at 1727 cm^−1^ observed in the pure PMMA, suggesting that *h-*BN was successfully coated on PMMA thus forming *h-*BN@PMMA (SBP) composite powder.

Figure 1(b) displays the characteristic peak of pure PU, *h-*BN/PU, and SBP/PU composites. In the pure PU spectrum, a peak located at 2917 cm^−1^ corresponds to the aliphatic (-CH-) asymmetric stretching mode, and a strong peak at around 1730 cm^−1^ correlates to the carbonyl absorption (C=O) stretching mode. The aromatic C-C stretching vibrations can be observed in the region around 1600 cm^−1^, and the peaks at 1530 cm^−1^ and 1230 cm^−1^ correspond to urea and urethane C-N stretching and N-H bending absorption^13,24^, respectively. The characteristic peak of *h*-BN can be found in *h*-BN/PU and SBP/PU spectrum, however, the intensity gets weaker due to the overlapping of PU and the additives peaks, which is also reported by other groups^25,26^.

Above results suggest that the inorganic fillers are not chemically linked with PU polymer and therefore the FTIR spectrum of *h*-BN/PU composite does not reveal the presence of new bonds. In addition, it was found that a strong characteristic peak appears at 801 cm^−1^ in the SBP/PU composite FTIR spectrum and this is due to the hydrogen interaction between the N-H group of PU and PMMA oxygen atoms^23,27^. Hence, the SBP composite is not only physically attached to the PU polymer but also attached to the PU matrix by chemical interactions during the manufacturing process.

**Figure 2:**
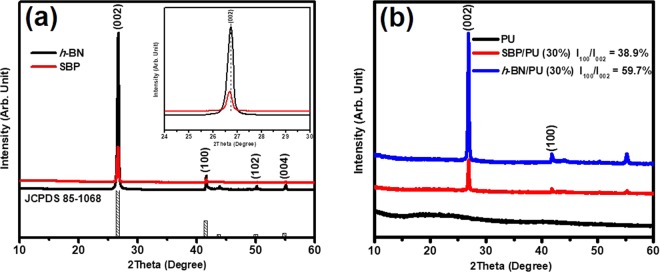
XRD patterns of (**a**) pure *h-*BN and SBP composite powders and the inset shows the scan curves of the pure *h-*BN and SBP (002) peak in the *θ*-2*θ* scan ranging from 2*θ* = 24 to 30°. (**b**) *h-*BN/PU and SBP/PU composites with 30 wt% filler loading, respectively.

Figure 2(a) shows the XRD pattern of the pure *h-*BN and SBP composite powders. The main peaks seen at 2*θ* = 27.11°, 41.95°, 50.50° and 55.35° can be assigned to the (002), (100), (102) and (004) planes respectively. From the diffraction data, it is clear that the above-mentioned peaks correspond to the typical hexagonal crystal *h-*BN structure, and no other hetero-phase can be detected (JCPDS Card no. 85–1068)^16,28^. From the main diffraction peaks of (002) and (004) plane as shown in Fig. 2(a), the diffraction peaks intensity of SBP composite powders is weaker than pure *h-*BN. This can be attributed to the exfoliation in the starting slurry of SBP composite powder due to mechanical mixing. Also, from the inset of Fig. 2(a), it reveals that the (002) diffraction peak of the SBP composite powder is slightly shifted towards the lower angle compared to that of pure *h-*BN. The shifting of the (002) diffraction peak could also be due to the exfoliation of *h-*BN sheets during the mechanical mixing process as verified by many several research works^29–31^. The pure PU demonstrates a broad diffraction peak at 20° as shown in the XRD pattern Fig. 2(b), and this is due to the amorphous structure of the polymer^32^. It was observed that both peaks of the *h-*BN/PU and SBP/PU composite have the same trend and no other obvious diffraction peaks were detected in both. These results imply that the crystal structure of *h-*BN particles is not affected by the processing method.

The degree of the orientation (δ) of BN sheets in the polymer matrix is one of the essential factors affecting the thermal conductivity, and it can be characterized by XRD analysis. The degree of orientation (δ) was calculated to realize the through-plane orientation degree of *h*-BN/PU composites and SBP/PU composites^15,33,34^. The Eq. (1) can be described as follows:

1 $${\rm{\delta }}=\frac{{I}_{(100)}}{{I}_{(100)}+{I}_{(002)}}\times 100 \% $$

where the *I*_(100)_ and *I*_(002)_ are the intensity of the (100) and (002) plane of *h-*BN, respectively. The (100) plane correlates to the vertically aligned *h-*BN sheets whereas the (002) plane represents the horizontal ones. As seen in the Fig. 2(b), the peaks located at 26.76° and 41.67° are the characteristic peaks of (002) and (100) planes. The δ value of SBP/PU composites and *h-*BN/PU composites are 38.9% and 59.7%, respectively with the δ value of SBP/PU composite 1.5 times lower than *h-*BN/PU composite indicating that the degree of orientation of *h-*BN in SBP/PU composite is higher than the *h-*BN/PU composite. It is worth to mention that it is challenging to prepare vertically aligned BN composite via traditional method^15,35^. However, through the process provided by this work, more vertically arranged *h*-BN structures can be created, which is beneficial to the powder dispersion in the composite material and to form the effective continuous heat conduction chains to provide more heat conduction paths.

**Figure 3:**
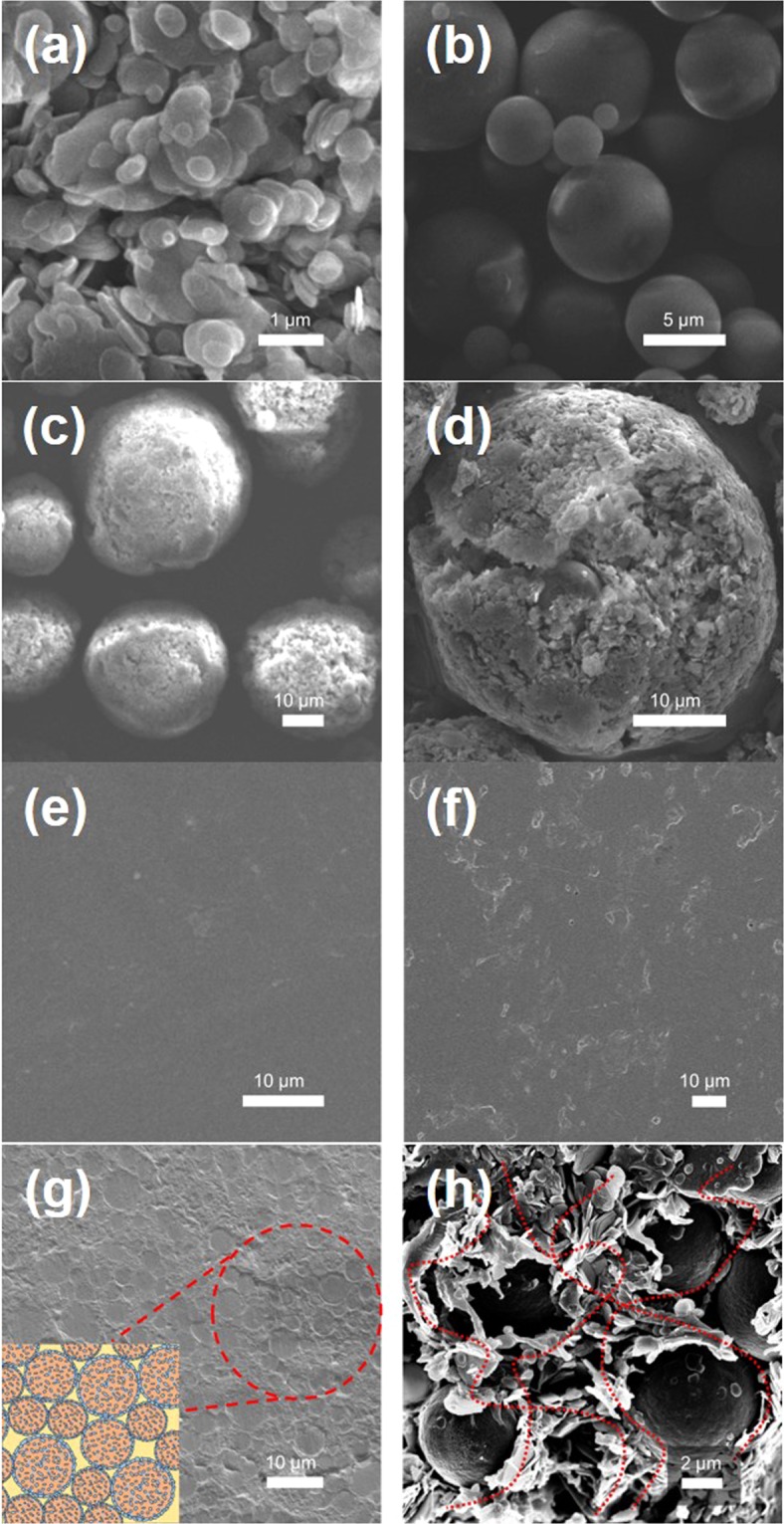
FE-SEM images of (**a**) pure *h-*BN, (**b**) PMMA, and (**c**) SBP composites, (**d**) cracked surface of (**c**) SBP spherical composite, (**e**) pure PU, (**f**) *h-*BN/PU, (**g**) SBP/PU composites with 20 wt% filler loading, and (**h**) rapid rupture surface of (**g**).

The surface morphology of the pure *h-*BN powder, PMMA powder and SBP composite was observed by field emission scanning electron microscopy (FE-SEM). Figure 3(a) shows the image of the pure *h-*BN powder. It can be seen that most of the *h-*BNs are stacked together and have a plate-like shape with average particle size of 1 *μ*m. The PMMA (average particle size = 4 *μ*m) powder has the spherical shape with the smooth surface as shown in Fig. 3(b), and this smooth surface is an important factor for the preparation of uniform and homogeneous coating. Figure 3(c) displays the surface morphology of SBP composite powder after spray drying. It can be observed that the composite powder has a rough surface which is due to the decoration of *h-*BN on the surface. Most of the composite powders have a spherical structure with good fluidity, and their particle sizes range from 10 and 40 *μ*m. To further distinguish the difference inside the composite powder, the powders were compressed between two parallel slides, and the results are shown in Fig. 3(d). It can be noted that the PMMA particles have relatively larger sizes and are located inside the particles, and the small-sized *h-*BN particles mainly form the shell of the composite powder. Moreover, the surface of the spherical composite powder possesses higher packing density which follows the same trend as reported in other studies. According to Brownian motion, small particles tend to move outward and form an outer shell, while large particles remain inside and form the core of the composite powder^36–38^.

The obtained SBP composite powder was further utilized to produce the SBP/PU composite by the solution mixing method. Figure 3(e–h) displays the cross-sectional images of the samples and the dispersion and structure distribution between *h-*BN, SBP powder and PU can also be observed in these figures. Figure 3(e,f) demonstrate the cross-section morphology of the pure PU and the *h-*BN/PU composite, respectively. From Fig. 3(e), the cross-section view of the pure PU is smooth and without any defects. The cross-section view of the *h-*BN/PU composite can be seen in Fig. 3(f), the surface is very rough and the dispersion of *h-*BN in the PU polymer is irregular. It can be due to the orientation of the filler cannot be controlled by the disperser stirring process, thereby forming the filler agglomeration. On the other hand, Fig. 3(g) shows the composite prepared from SBP spherical composite powder. After stirring in the same manner, a uniform dispersion and tight connection with the PU polymer is formed (marked in red), and this increases the number of heat conduction path that assists in accelerating the phonon to transmit in the direction of the heat flow^39^. Particle dispersion and the conducting path of each composite are represented using the schematic diagram shown in the inset of Fig. 3(g). Figure 3(h) is the cross-section view of the sample that underwent the rapid rupture after immersing in the liquid nitrogen. This finding proves that the spherical PMMA microspheres inside the SBP composite powder encapsulated by the *h*-BN shell, allows the heat flow to transfer directly through the shell structure of *h*-BN and form a tight and continuous heat flow path (mark in red). As a result, the construction of a continuous filler network with high thermal conductivity can be expected^40–42^.

**Figure 4:**
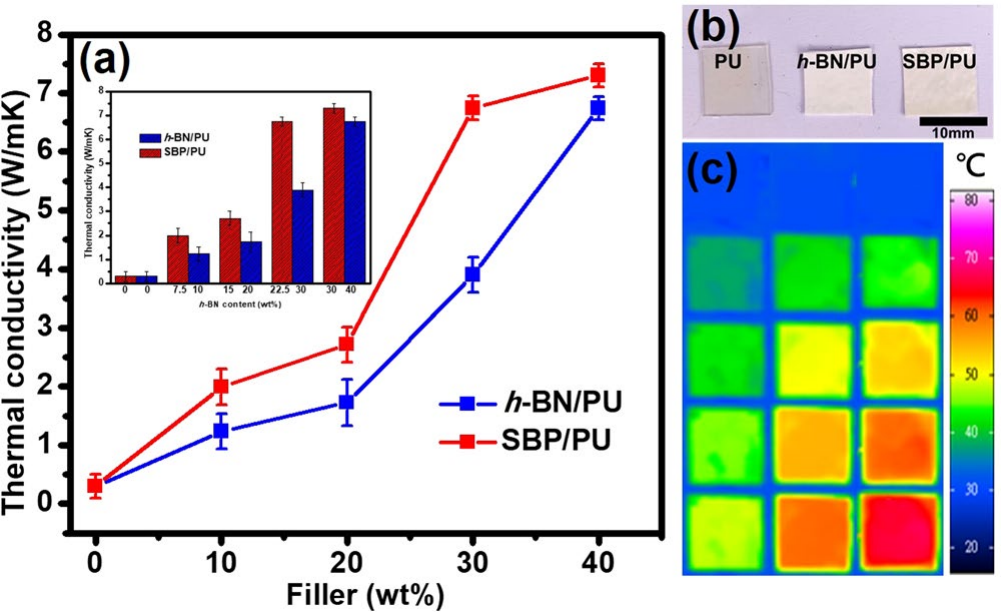
(**a**) Thermal conductivity of *h*-BN/PU and SBP/PU composites versus filler loading (wt%), and the inset shows the thermal conductivity of *h*-BN/PU and SBP/PU composites versus different *h*-BN content (wt%). (**b**) Photographs of the pure PU, *h*-BN/PU, and SBP/PU composite. (**c**) Infrared thermal images of pure PU, *h*-BN/PU and SBP/PU composites with 30 wt% filler concentration.

Figure 4(a) shows the thermal conductivity of PU composites with different filler loadings of SBP and *h-*BN prepared by using the disperser under the same conditions. The results indicate that the thermal conductivity of all the composites increases with increasing the content of the filler. However, the trend of thermal conductivity of all samples is nonlinear. The threshold for percolation can be found to exhibit between 10 wt% and 20 wt% of the filler loadings because the polymer matrix would be interposed between the adjacent fillers under low filler loading and it cannot fully contact with each other, therefore, the phonon can be scattered which further increases the interface thermal resistance, leading to poor heat conduction^25^. On the other hand, with increase in the filler loading, the trend of thermal conductivity shows a sharp rise at 20 wt%. The increasing number of fillers contact with the adjacent ones, forming a densely packed structure that facilitates phonon transfer in a continuous thermal network^43,44^. Further, with the filler loading increased from 30 wt% to 40 wt%, the increasing trend of the thermal conductivity of the SBP/PU composite gradually slows down. This finding means that the adjacent spherical SBP fillers in SBP/PU composites are in direct contact with each other and the number of contacts has almost reached the saturation at 40 wt% of the filler loading^25^. For industrial application, it is worth to figure out the real content of *h*-BN in the SBP composite. Therefore, we compared SBP/PU and *h-*BN/PU composites with the same *h*-BN content by calculating the ratio of *h*-BN loading in the SBP/PU composite as shown in the inset of Fig. 4(a). The highest thermal conductivity of 7.302 W m^−1^ K^−1^ was observed in the PU composites with SBP filler of 40%, but the actual content of *h*-BN in SBP/PU was 30%. Despite the lower loading of the *h*-BN filler in the SBP/PU composite, the significant improvement in the thermal conductivity of SBP/PU can still be seen. These results indicate that the SBP can tightly stack the *h*-BN powder in the PU matrix, forming the continuous thermal network structures and effectively improving the interfacial interaction.

Figure 4(b) presents the photographs of the pure PU, *h-*BN/PU, and SBP/PU composites. The pure PU exhibits high transparency, while the *h-*BN/PU and SBP/PU composites are in white. To directly demonstrate the heat transfer capability of *h-*BN/PU and SBP/PU composite materials, the samples were heated through an electric hot plate, and the temperature response was recorded by the infrared thermography. Before the measurement, all the samples were maintained in the same condition in order to avoid surface roughness, thickness, and geometry of the samples to cause the heat loss. Figure 4(c) shows the infrared thermal images of pure PU, 30 wt% *h-*BN/PU and 30 wt% SBP/PU composites. The surface temperature changes with the increase in the heating time and the color of the sample gradually changes from blue to red. The results indicate that the addition of fillers brings the significant difference in the PU by enhancing its thermal conductivity. In addition, SBP/PU composites prepared by the spray drying process exhibit better heat transfer capability than *h-*BN/PU composites under the same time conditions. It can thus be confirmed that our method can effectively disperse the filler and construct the thermal network to improve the thermal conductivity of the composites.

## Conclusion

In summary, the spherical *h*-BN composite powders (SBP) can be effectively utilized in optimizing the thermal conductivity of PU composites by simple mechanical mixing and spray drying processes. Compared to *h*-BN/PU composites prepared with the same *h*-BN loading, the SBP/PU composite possesses significantly higher thermal conductivity. The SBP/PU composite with 30 wt% of *h*-BN loading, it can reach a high thermal conductivity of 7.3 Wm^−1^ K^−1^. Our method can improve the thermal conductivity of the *h*-BN/PU composite by two-fold with the same *h*-BN loading. The peak at 801 cm^−1^ seen in the FTIR spectrum confirms the chemical interaction between the SBP composite powder and the PU matrix. SBP composite powder effectively increases the orientation of *h*-BN in the PU matrix by the method provided in this work. SBP spherical composite powders also exhibit good dispersion and well-ordered continuous *h*-BN thermal conductive networks in the PU polymer matrix. We have successfully provided with the application strategy for developing the low filler-loaded high thermal conductivity polyurethane composite with 3-dimensional continuous network structure of *h*-BN.

## Material and Methods

### Materials

The *h-*BN powders with particle size ranging from 1–2 *μ*m were provided by National Nitride Technologies Co., Ltd. (Taichung, Taiwan). Polymethyl methacrylate (PMMA, M_W_ = 22,000 g mol^−1^, Density: 1.19 g cm^−3^, supplier data) powders with the particle size of 5 *μ*m were purchased from Eternal Materials CO., Ltd. (Kaohsiung, Taiwan). Waterborne Polyurethane (PU, M_W_ = 240,000 g mol^−1^, Density: 1.18 g cm^−3^, supplier data) was provided by Win-Z Technology Co., Ltd (Taichung, Taiwan). Toluene (C_7_H_8_) was provided by Aencore Chemical Co., Ltd (Australia). All chemicals used in the experiment are of analytical grade and without any further purification.

### Preparation of *h-*BN@PMMA (SBP) spherical composite

**Figure 5:**
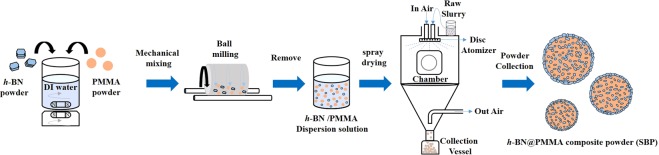
Schematic diagram portraying the steps involved in preparation of spherical *h*-BN@PMMA composite powder (SBP).

Mechanical mixing and spray drying were used in this process. The *h-*BN powder was uniformly mixed with PMMA to form a slurry. Both the powders were added to DI water (10 L) in the ratio of 3:1 (2.8 kg). After agitation with the magnetic stirrer, the components were thoroughly mixed by the vertical ball mill to obtain the uniform *h*-BN/PMMA dispersion solution. The above-formed dispersion solution was added to the spray drying system (CNK SDDNO-3, IDTA Machinery Co., Ltd., Taipei, Taiwan). The following process parameters were described as below: the temperature in air and out of air was set at range of 200 to 220 °C and 80 to 120 °C, the temperature of chamber was set at the range of 90 to 130 °C, press air was set at 1.5 kg/cm^2^, disc rotation speed was 30,000 rpm, and the feed rate was fixed at 3 kg/h. After the mixture was treated by the spray drying process, the spherical *h-*BN@PMMA composite (SBP) was obtained. The experimental procedure is shown in Fig. 5.

### Composite preparation

The PU composite was prepared by using solution mixing technology. Firstly, 20 g of PU was dissolved in 34.6 ml of toluene, and different proportions of SBP were added into the PU solution. Then, the mixture was stirred by the disperser (T25 digital, IKA Works, Germany) for 30 mins to obtain the uniform polymer mixture. After mixing, the homogeneous mixture was poured into the TeflonTM wells and dried in a vacuum oven at 60 °C for 24 hours to remove the solvent. The SBP/PU composite material was then cut into the size of 1 × 1 cm^2^ after drying.

The purpose of this study was to evaluate the consistency and continuity of the filler, so the same mixing method was used to prepare different proportions of *h-*BN/PU composites for the control sets. The dried SBP/PU and *h-*BN/PU samples were all white films. 10 wt% 20 wt% 30 wt% and 40 wt% of filler in SBP/PU and *h-*BN/PU composite were prepared through adding different proportions of filler during the process.

### Characterization

The crystal structure of fillers and composite materials were characterized by X-ray diffraction (EMPYREAN, Panalytical Co., Ltd, Netherland) using Cu Kα radiation (λ = 1.54 Å) in range of 2*θ* = 10° − 60° with step size and the scan speed of 0.05° and 2°/min, respectively. Fourier transform infrared spectrometer (JASCO FT/IR-4150, Japan) was used to analyze the chemical structure of thermally conductive PU composites and fillers and the spectrum wavenumber ranged from 400 to 4000 cm^−1^. Top view and cross-section view microstructures of the prepared BN particles and composites were observed using secondary electron (SEI) method through field emission scanning electron microscope (FE-SEM, JSM-7610F, JEOL, Japan). The thermal conductivity of the composite was measured using the stable state method by thermal impedance tester (LW9053, Long Win Science and Technology Co., Ltd., Taoyuan, Taiwan) according to the ASTM D-5470-01 standard^20,45,46^. Finally, Infrared thermography (IR-TCM HD, Jenoptik AG, Germany) was used to record the heat transfer surface temperature of composite films.
